# A Case of COVID-19-Associated Focal Segmental Glomerulosclerosis

**DOI:** 10.7759/cureus.37547

**Published:** 2023-04-13

**Authors:** Anish D Thomas, Robert Trainor, Zackery Sheingold, Mark Samarneh

**Affiliations:** 1 Internal Medicine, Riverside Health System (St John's Riverside Hospital), Yonkers, USA; 2 Internal Medicine, Lake Erie College of Osteopathic Medicine, Erie, USA; 3 Internal Medicine/Nephrology, Riverside Health System (St John's Riverside Hospital), Yonkers, USA

**Keywords:** secondary glomerulopathy, hiv diseases, kidney disease, covid19, focal segmental glomerular sclerosis, glomerulosclerosis

## Abstract

This case report details a 43-year-old female diagnosed with the collapsing variant of focal segmental glomerulosclerosis (FSGS) post-infection with coronavirus disease 2019 (COVID-19). The patient contracted COVID-19 after returning from a trip to Florida and initially presented to the emergency department with gastrointestinal symptoms. Thereafter, the patient was diagnosed with COVID-19 and was admitted for acute kidney injury and worsening COVID-19 infection. FSGS is a glomerulopathy that consists of glomerular scarring that leads to nephrotic syndrome, secondary to podocyte effacement. FSGS has many causes, as well as distinct variants, but is noted to have an association with some viruses, most notably HIV and cytomegalovirus (CMV). Although the association between FSGS and HIV or CMV is well established, the evidence is minimal in regard to other viruses. This case report serves to highlight the potential association of COVID-19 with FSGS.

## Introduction

Focal segmental glomerulosclerosis (FSGS) is a glomerulopathy consisting of glomerular scarring that leads to a nephrotic syndrome secondary to podocyte effacement. It has an incidence of seven per million and an estimated prevalence of 4% [1]. FSGS has been linked to multiple causes including viral infection with viruses including, but not limited to, HIV, parvovirus B-19, hepatitis B and C, and cytomegalovirus (CMV) [2]. The collapsing variant of FSGS, such as in our patient, has been frequently linked to viral causes, possibly through viral infection of visceral cells in the glomerulus. Collapsing FSGS is categorized as a variant of FSGS in which there is segmental or global glomerular collapse with visceral epithelial cell swelling, hyperplasia with hyaline droplets, and extensive tubulointerstitial inflammation [3]. In this report, we will discuss the case of a 43-year-old female diagnosed with collapsing FSGS after infection with severe acute respiratory syndrome coronavirus 2 (SARS-CoV-2).

## Case presentation

A 43-year-old female with a past medical history of obesity with a BMI of 46 presented to the emergency department for the second time with worsening symptoms after being diagnosed with coronavirus disease 2019 (COVID-19). She had no prior history of kidney disease or asthma, and had normal creatinine of 1.0 in June 2021 at the primary care office. She had been seen in the emergency department one day prior for gastrointestinal symptoms and ultimately tested positive for COVID-19. The patient had returned on a flight from Florida to New York that day and was concerned she had food poisoning. After treatment with intravenous fluids and electrolytes, she was discharged home. Her COVID-19 results were still pending, but her blood work and renal function were all normal. The following day, the patient returned to the emergency department after results showed she was positive for COVID-19. This time, she noted worsening abdominal pain, nausea, vomiting, and diarrhea for a total of six days as well as new onset urinary hesitancy. Her vital signs including blood pressure and heart rate were stable on admission.

**Figure 1:**
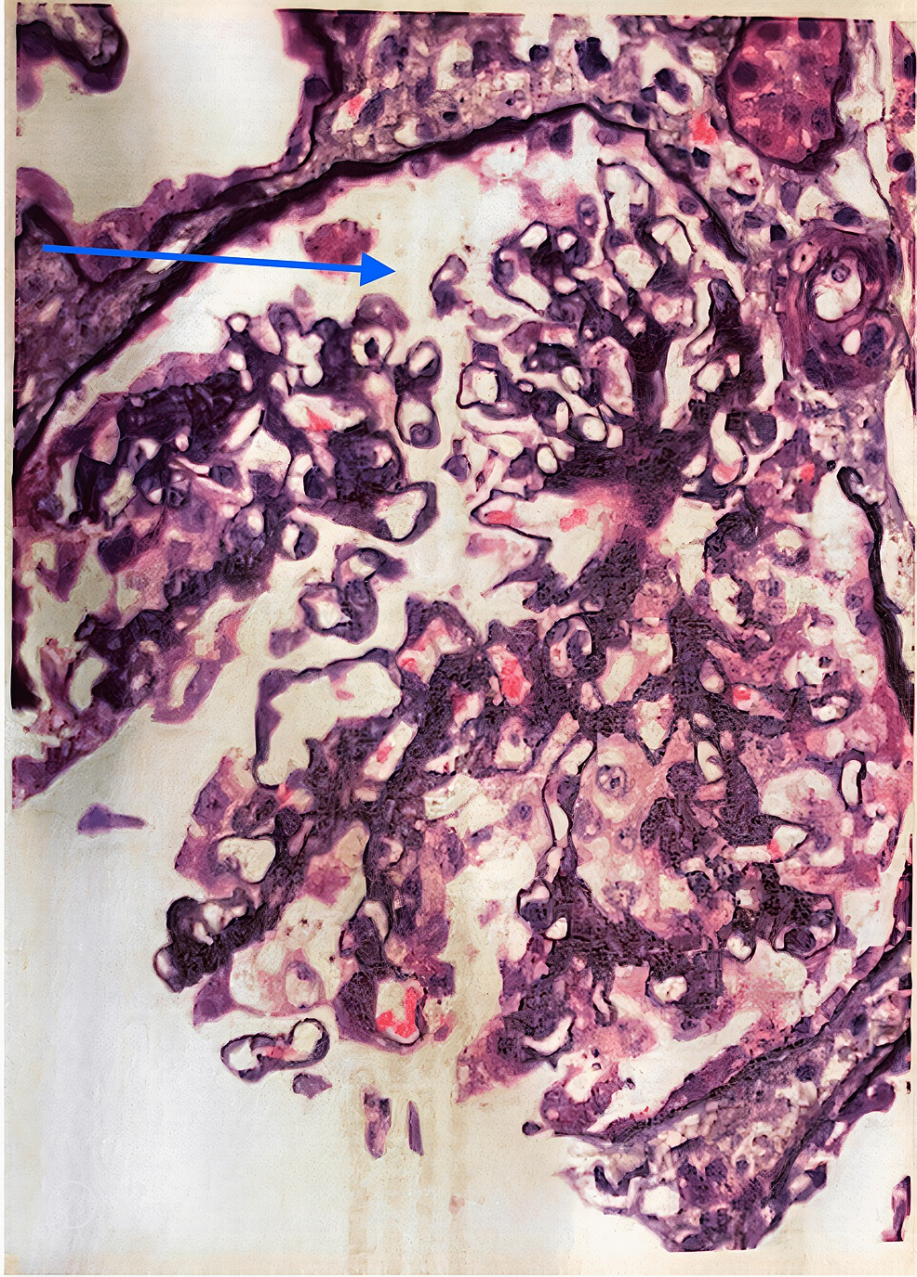
Collapsing FSGS, mild (collapsing lesions involving two of 13 glomeruli, absence of significant tubulo-interstitial scarring) FSGS: focal segmental glomerulosclerosis

**Figure 2:**
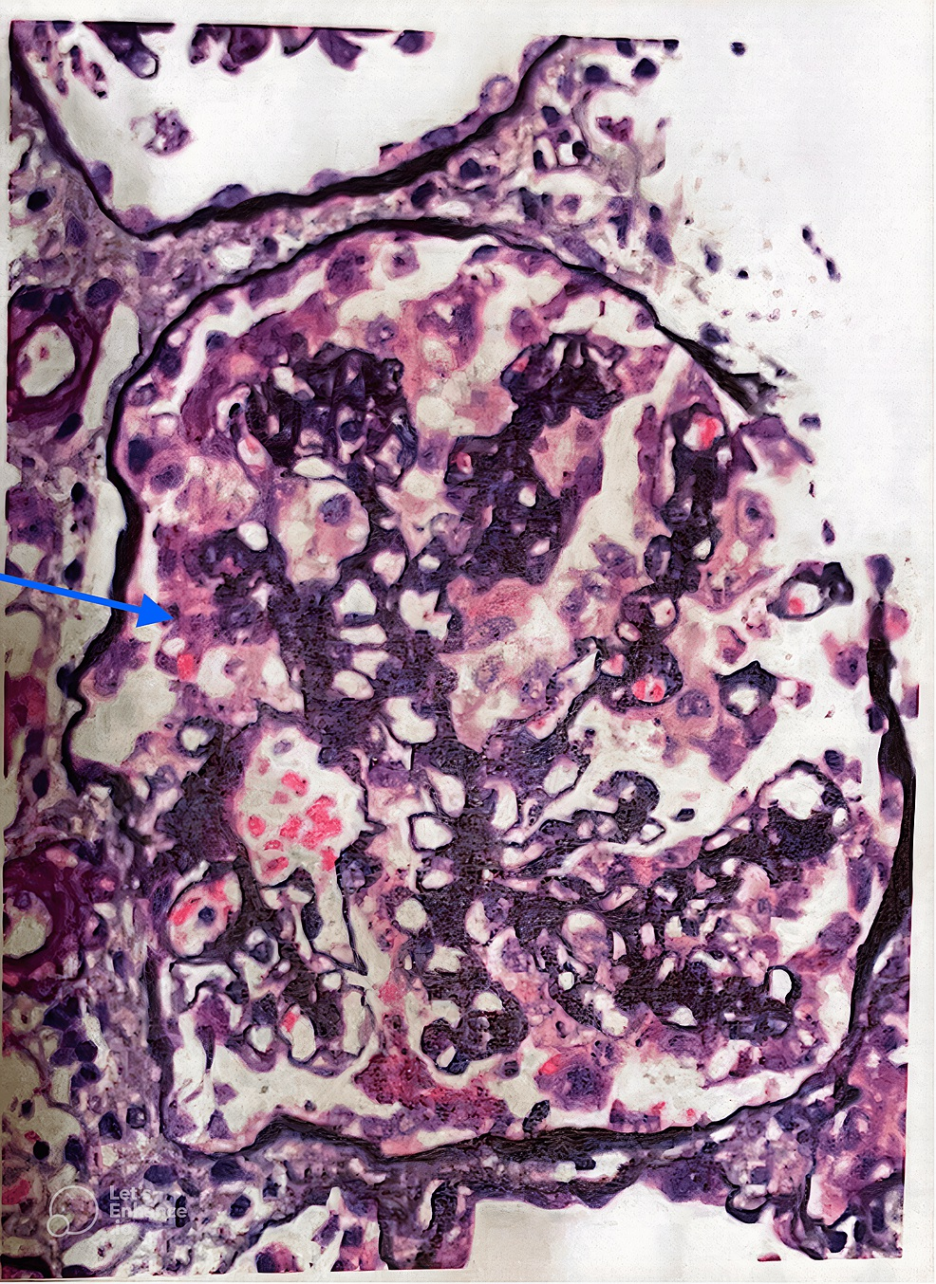
Endocapillary hypercellularity of the cellular variant

She was administered two liters of normal saline, 2mg of morphine for pain, and 4mg of ondansetron for nausea. A Foley catheter was also placed, due to her inability to produce any urine after a few hours. The result of her urinalysis was remarkable for proteinuria and hematuria with casts as shown in Table 1. Her creatinine had increased within 24 hours since admission also shown in Table 1. Therefore, the medical team was consulted, and the patient was admitted for suspected acute kidney injury (AKI) secondary to COVID-19 infection. On day 2 of admission, a renal ultrasound impression showed slightly increased echogenicity of the renal cortices consistent with medical nephropathy, but visualization was limited due to body habitus. On day 6 of admission, with the recommendation of the nephrologist, the patient underwent a renal biopsy, which was sent to Columbia University for consultation. Thereafter, the pathology report returned with a diagnosis of either primary or secondary collapsing FSGS, likely due to a viral cause such as COVID-19 infection or HIV infection. Therefore, due to the involvement of only two out of 13 glomeruli sampled as shown in Figure 1 and Figure 2, the prognosis was favorable. 

**Table 1:** Laboratory value highlighting renal function and urinalysis results LPF= low power field

	Units	On admission	24 hours after admission	Reference ranges
Creatinine	mg/dL	1.0	2.3	0.7-1.3
Protein	mg/dL	4+		Negative (<20)
Blood	mg/dL	3+		Negative (<0.02)
Cast	LPF	Present		None

## Discussion

FSGS accounts for 40% of nephrotic syndromes in adults and 20% of nephrotic syndromes in children, and is considered the most common primary glomerular disease resulting in end-stage renal disease in the United States [1]. The collapsing variant is one of five histological variants of FSGS and appears to be more often linked with viral causes than the other subtypes, specifically HIV [4]. Although the association of collapsing FSGS with HIV and CMV is relatively established, the COVID-19 pandemic has provided new cases of collapsing FSGS in patients without either of the aforementioned viruses and only infection with the novel SARS-CoV-2 virus. A systematic review by Cancarevic et al. (2022) suggests the development of nephrotic syndrome, especially FSGS, in patients infected with COVID-19 [5]. Another study by Shetty et al. (2021) analyzed the relationship between COVID-19-associated glomerular disease, in which they found that the glomerular disease caused by COVID-19 was either FSGS, podocytopathy, or both [6]. Multiple case reports including a report by Malik et al. (2020) also suggest a causal relationship between COVID-19 infection and FSGS [4]. Considering all the previous studies, and a negative viral panel in our patient, including HIV, Hepatitis B and C, and CMV, suspicion was strengthened for her glomerulopathy being a result of her COVID-19 infection. 

The collapsing variant of FSGS is defined by the Columbia classification as the presence of segmental capillary tuft collapse (wrinkling and folding) in at least one glomerulus [7]. In the biopsy sample from our patient as shown in Figure 1, two out of 13 glomeruli were affected, thus meeting the Columbia criteria for diagnosis of the collapsing variant. The difference between the collapsing variant and traditional cellular FSGS is the implosive wrinkling and retraction of the glomerular basement membrane, along with endocapillary hypocellularity as opposed to the endocapillary hypercellularity of the cellular variant as shown in Figure 2 [8]. All these findings lead to several plausible causes for this patient's FSGS. It is likely that the COVID-19 infection induced an autoimmune response, similar to that seen in the lungs, leading to the worsening of an undiagnosed or underlying FSGS or the development of a new FSGS picture. While there are no specific treatments for the collapsing variant, the mainstays of treatment are those currently used for non-collapsing variants, which include steroids and immunosuppressive agents. In the case of this patient, she was treated with steroids and followed up outpatient. 

With minimal research into the viral causes of FSGS, this case serves to revisit this potential association while showcasing an emerging link with COVID-19. AKI has been noted in COVID-19 patients more frequently in the last few years, with some studies suggesting nearly 45% of COVID-19 patients that are admitted to the ICU. A large portion of these patients eventually requires kidney replacement therapy, which results in an increased risk of mortality [9]. COVID-19-induced FSGS has been reported in several case reports in recent years as well, some of which noted the relationship between *APOL1* gene carriers and increased risk of FSGS in COVID-19 [9]. There are a few case reports also suggesting the role of *APOL1* gene testing in African Americans due to the increasing number of similar case reports showing the increased prevalence of this gene in this population, leading to COVID-19-induced FSGS [10,11]. Reporting these cases and identifying this relationship allows more medical providers to be aware of this essential link and can help in both prompt surveillance after infection with COVID-19 and early treatment if diagnosed.

## Conclusions

Collapsing FSGS is a variant of FSGS that is responsible for a large percentage of nephrotic syndromes and can ultimately lead to end-stage renal disease in some patients. While the incidence of collapsing FSGS has been linked with some viruses, this association has yet to be proven as the evidence is inconclusive. However, with the ongoing COVID-19 pandemic and new cases arising in COVID-19-infected patients, it is important to consider that the novel coronavirus, SARS-CoV-2, could potentially be a common cause of AKI secondary to collapsing FSGS. Remaining alert to patients presenting with early signs of AKI can help to distinguish this uncommon, yet potential complication of COVID-19 infection moving forward.

## References

[1] Sprangers B, Meijers B, Appel G (2016). FSGS: diagnosis and diagnostic work-up. Biomed Res Int.

[2] Dettmar AK, Oh J (2016). Infection-related focal segmental glomerulosclerosis in children. Biomed Res Int.

[3] Weiss MA, Daquioag E, Margolin EG (1986). Nephrotic syndrome, progressive irreversible renal failure, and glomerular ‘collapse’: a new clinicopathologic entity?. Am J Kidney Dis.

[4] Malik IO, Ladiwala N, Chinta S, Khan M, Patel K (2020). Severe acute respiratory syndrome coronavirus 2 induced focal segmental glomerulosclerosis. Cureus.

[5] Cancarevic I, Nassar M, Medina L (2022). Nephrotic syndrome in adult patients with COVID-19 infection or post COVID-19 vaccine: a systematic review. Cureus.

[6] Shetty AA, Tawhari I, Safar-Boueri L (2021). COVID-19-associated glomerular disease. J Am Soc Nephrol.

[7] Chandra P, Kopp JB (2013). Viruses and collapsing glomerulopathy: a brief critical review. Clin Kidney J.

[8] Mubarak M (2012). Collapsing focal segmental glomerulosclerosis: current concepts. World J Nephrol.

[9] Craig T, Ansari M, Foster P, Abdelgadir Y, Abdelghani A, Jha P (2022). A case of COVID-associated nephropathy (COVAN). Cureus.

[10] Magoon S, Bichu P, Malhotra V, Alhashimi F, Hu Y, Khanna S, Berhanu K (2020). COVID-19-related glomerulopathy: a report of 2 cases of collapsing focal segmental glomerulosclerosis. Kidney Med.

[11] Nasr SH, Kopp JB (2020). COVID-19-associated collapsing glomerulopathy: an emerging entity. Kidney Int Rep.

